# Editorial: Interventional Strategies for Enhancing Quality of Life and Health Span in Older Adults

**DOI:** 10.3389/fnagi.2020.00253

**Published:** 2020-09-11

**Authors:** Mario Bernardo-Filho, Michael G. Bemben, Redha Taiar, Borja Sañudo, Trentham Furness, Brian C. Clark

**Affiliations:** ^1^Departamento de Biofísica e Biometria and Policlínica Piquet Carneiro, Rio de Janeiro State University, Rio de Janeiro, Brazil; ^2^Department of Health and Exercise Science, University of Oklahoma, Norman, OK, United States; ^3^Groupe de Recherche en Science pour l'Ingénieur (GRESPI), Université de Reims Champagne-Ardenne, Reims, France; ^4^Departamento de Educación Física Y Deporte, Universidad de Sevilla, Seville, Spain; ^5^Faculty of Health Sciences, Australian Catholic University, Sydney, NSW, Australia; ^6^Department of Biomedical Sciences, Ohio Musculoskeletal and Neurological Institute, Ohio University, Athens, OH, United States

**Keywords:** older adults, interventional strategies, quality of life, health span, neuroscience of aging

Across our lifespan a wide range of factors can influence human health and well-being. Normal aging is associated with the accumulation of deleterious and undesirable changes, often resulting in an increased risk of co-morbid diseases and premature mortality. Geriatric syndromes, including frailty, sarcopenia, and cognitive impairment, are increasing worldwide and there is a growing need for cost-effective geriatric assessment and management strategies that can be implemented in a large number of settings. Technologies are playing an important role for the identification of certain geriatric syndromes, especially for healthcare professionals in the primary care setting. For example, rapid and feasible tools (e.g., mobile applications) such as the Rapid Geriatric Assessment (RGA) are now widely used for this purpose (Merchant et al.). While the development of pharmacological interventions have resulted in major advances in the management of numerous age-related conditions, there is still an urgent need to offer and develop simple and effective non-pharmacological interventions with known limited side effects to robustly improve quality of life (QoL) and independence in older adults. In particular, increased focus on interventional strategies that enhance both physical and cognitive function in the elderly are critically needed. It was historically believed that the neural substrates that determined motor and cognitive function were discrete; however, more recent conceptual frameworks, such as the concepts such as the “neural reuse theory” suggests it is quite common for neural circuits established for one purpose (e.g., movement) to be exploited, recycled, and/or redeployed during evolution or normal development and be put to different uses (e.g., cognition), often without losing their original functions (Anderson, 2007, 2010). Thus, the neural reuse theory offers an interesting perspective on the degree of localization of cognitive function, and suggest that portions of the brain that are involved in motor function are also involved in cognitive function (Anderson, 2007, 2010).

Physical exercise-based strategies have been established for the improvement of detrimental changes associated with aging, even in animal models (Macit et al.). Unfortunately, most older adults do not exercise regularly (Tavoian et al.) and some of them are reluctant to even initiate a program of increased physical activity; therefore, other strategies need be used to prevent the functional decline in the elderly (Valenzuela et al., 2019). Different exercise programs including aerobic training (even at high intensities), strength training or mixed methods, have been suggested in order to improve both cardiorespiratory and muscle function in older adults (Tavoian et al.).

The functional decline described above can affect the ability to perform activities of daily living in older adults (Malmstrom et al., 2016; Sañudo et al., 2020) as a result of deterioration in the sensory systems (i.e., vestibular, visual, somatosensory), neurocognitive networks/system, and the musculoskeletal system. Consequently, many older adults are at risk of falling during daily life. One study in this issue (Dunsky) highlighted different exercise-based programs used in fall prevention. These programs challenge the sensory, cognitive, and musculoskeletal systems while addressing balance constraints, such as orientation in space, changes in direction, and the speed or height of the center of mass during static and dynamic situations resembling ADL. The author suggested that among older adults, programs that include a combination of dual-task, function-oriented challenges while controlling balance stimulates the sensory and neuromuscular control mechanisms, and have been found to improve static and dynamic stability, as well as a number of aspects in QoL (Dunsky). In another study, evidence was presented that traditional dance programs have the potential to improve the physical fitness and wellbeing of the elderly (Douka et al.). In this study, a 32 week dance intervention (2 times per week, for 75 min per session) using different intensities, was reported to improve physical fitness (e.g., handgrip strength, chair stand, and indices of flexibility) and static balance. Other articles in this issue: (1) assessed the effects of moderate-intensity treadmill aerobic training (8 weeks) on redox status and inflammatory biomarkers and motor performance in rats with knee osteoarthritis, and noted that motor performance in all joint function tests was improved and the inflammatory biomarkers were reduced with exercise (Martins et al.); and (2) evaluated the effects of adding whole body vibration to squat training on muscle strength and the plasma levels of brain-derived neurotrophic factor (BDNF) in elderly woman with knee osteoarthritis (Simão et al.). Here, improvements in lower limb muscle performance associated with neuromuscular adaptations (BDNF plasma levels) were observed. Lastly, one article described the protocol for an ongoing randomized control trial investigating the comparative effectiveness for three different pragmatic exercise intervention approaches (high-intensity interval cycle training vs. moderate, continues cycle training vs. progressive resistance exercise) to improve a comprehensive battery of physical fitness parameters (e.g., maximal oxygen consumption, muscular power and strength, 6-min walk distance, fatigue resistance) and body composition (e.g., fat mass and lean mass) (Tavoian et al.).

On the other hand, *cognitive training* was also reported to successfully promote healthy aging (Alnajjar et al.). Recent studies suggest that computer-based cognitive interventions could be effective at administering cognitive training for older adults (Alnajjar et al.). In the same line, Weng et al. explored the effects of cognitive training on working memory in older adults with mild cognitive impairment. The authors reported that the training effects on working memory could also be transferred to other untrained areas (such as executive function). Moreover, similar cognitive training strategies were also tested to improve car driving skills of older adults (Nouchi et al.). After only 6 weeks of training, cognitive functions such as processing speed, inhibition, and vigor–activity mood, were enhanced in healthy older people.

The acquisition and retention of motor skills is necessary for everyday functioning in the elderly and may be critical in the context of motor rehabilitation. Studies indicate that motor training closely followed by sleep may result in better engagement of procedural (“how to”) memory consolidation processes in the elderly. Intriguing findings from Gal et al. suggest that evening (6–9 p.m.) training (multi-session motor practice program over 10 sessions across 3–4 weeks) enhances motor skill learning in older adults. The results are in line with the notion that motor training preceding a sleep interval may be better consolidated into long-term memory in the elderly, and thus result in lower forgetting rates (Gal et al.).

Other alternative strategies, such as acupuncture, were also reported to improve brain health, with both neural and vascular mechanisms being described (Sun et al.). Other newer strategies, such as exploratory behavior and responsiveness to novelty were reported to play an important role in maintaining cognitive function in older adults. In this study, Behforuzi et al. investigated the short-term test-retest reliability of event-related potential (ERP) and behavioral responses to novel stimuli in cognitively normal older adults. Their findings suggested that older adults may have a characteristic way of processing novelty that appears resistant to transient changes in their environment or internal states, which can be indexed during a single testing session. The establishment of reliable measures of novelty processing will allow investigators to determine whether proposed interventions have an impact on this important aspect of behavior (Behforuzi et al.).

In summary, in this special issue a broad range of non-pharmacologic, and relatively simple and pragmatically implementable approaches are presented that aim to increase life expectancy, promote health and independence, and/or improve QoL for the elderly via interventional strategies targeting, in part, the nervous system.

## Author Contributions

MB-F, BS and TF prepared the first version. All authors listed have made a substantial with direct and intellectual contribution to the final version that was approved for publication. In addition, MB and BC reviewed the English language.

## Conflict of Interest

In the past 5-years, BC has received research funding from NMD Pharma, Regeneron Pharmaceuticals, Astellas Pharma Global Development, Inc., and RTI Health Solutions for contracted studies that involved aging and muscle related research. In the past 5-years, BC has received consulting fees from Regeneron Pharmaceuticals, Zev industries, and the Gerson Lehrman Group for consultation specific to age-related muscle weakness. BC is a co-founder with equity of AEIOU Scientific. The remaining authors declare that the research was conducted in the absence of any commercial or financial relationships that could be construed as a potential conflict of interest.
